# Empirical validation of QUEST+ in PSE and JND estimations in visual discrimination tasks

**DOI:** 10.3758/s13428-022-02001-4

**Published:** 2022-12-20

**Authors:** Adrien Paire, Anne Hillairet de Boisferon, Céline Paeye

**Affiliations:** Université Paris Cité, Vision Action Cognition, F-92100 Boulogne-Billancourt, France

**Keywords:** Psychophysics, QUEST+, Constant stimuli, Staircase, Visual discrimination

## Abstract

One of the most precise methods to establish psychometric functions and estimate threshold and slope parameters is the constant stimuli procedure. The large distribution of predetermined stimulus values presented to observers enables the psychometric functions to be fully developed, but makes this procedure time-consuming. Adaptive procedures enable reliable threshold estimation while reducing the number of trials by concentrating stimulus presentations around observers’ supposed threshold. Here, the stimulus value for the next trial depends on observer’s responses to the previous trials. One recent improvement of these procedures is to also estimate the slope (related to discrimination sensitivity). The Bayesian QUEST+ procedure (Watson *Journal of Vision, 17*(3), 10, 2017), a generalization and extension of the QUEST procedure, includes this refinement. Surprisingly, this procedure is barely used. Our goal was to empirically assess its precision to evaluate size, orientation, or temporal perception, in three yes/no discrimination tasks that increase in demands. In 72 adult participants in total, we compared points of subjective equivalence (PSEs) or simultaneity (PSSs) as well as discrimination sensitivity obtained with the QUEST+, constant stimuli, and simple up-down staircase procedures. While PSEs did not differ between procedures, sensitivity estimates obtained with the 64-trials QUEST+ procedure were overestimated (i.e., just-noticeable differences, or JNDs, were underestimated). Overall, agreement between procedures was good, and was at its best for the easiest tasks. This study empirically confirmed that the QUEST+ procedure can be considered as a method of choice to accelerate PSE estimation, while keeping in mind that sensitivity estimation should be handled with caution.

**Supplementary Information:**

The online version contains supplementary material available at 10.3758/s13428-022-02001-4.

## Introduction

The purpose of psychophysics is to accurately and objectively evaluate the relationship between physical dimensions of stimuli and perceptual responses. During psychophysical procedures, stimuli varying along a given physical dimension are presented to observers who have to indicate whether they perceive the stimuli (detection), or whether they differ from a reference stimulus (discrimination).

One of the most straightforward psychophysical methods is the method of constant stimuli (CS). In this procedure, the distribution of stimulus values covers a large range of values and is specified before the experiment. The advantage of this method is that the psychometric function (i.e., observers’ responses as a function of stimulus values) can be fully developed. Thresholds (i.e., stimulus values leading to a given percentage of determined levels of detection or discrimination) and slopes (i.e., the rate of change in performance given stimulus values) can thus be determined accurately. Nevertheless, there are also many trials that are far from the observers’ threshold and therefore non-informative. This makes the CS procedure time-consuming and requires patience from both observers and experimenters.

Adaptive procedures have been designed and developed to increase measurement efficiency, i.e., to obtain valid, precise, and reliable threshold estimation while minimizing experimental time and observers’ efforts. They do not require responses to a complete set of physical stimulus values, but concentrate stimulus presentations around observers’ threshold. We will not describe these procedures extensively; the reader is referred to the excellent reviews by Treutwein (1995) and Leek (2001) on their historical and quantitative developments.

Among adaptive procedures, nonparametric methods, called staircase procedures, use responses to the previous trial or a sequence of trials, within an adaptive track, to select the stimulus physical value for the next trial. No assumption is made about the shape of the psychometric function, except that it is strictly monotonic. Stimulus placement rules along the array, step sizes, and termination rules differ according to the specific method used. For instance, in a simple up-down staircase procedure, the stimulus value is changed by a fixed step size after each response. Stimulus value adjustments will remain in the same direction unless the observer changes response category (reversal) (e.g., Cornsweet, 1962). The procedure ends after a fixed number of reversals. In this specific case, the track targets a 50% performance level (i.e., the threshold) on the psychometric function. Because of their simplicity and flexibility, adaptive staircase procedures are widely used in many laboratories.

In adaptive parametric methods, the general form of the underlying psychometric function must be assumed beforehand, and used as a template. After each trial or block of trials, values of one (or more) parameters of the psychometric function are estimated, typically with a maximum-likelihood fitting algorithm. The new parameter estimates, which are refined over the course of the experiment, are used to determine the most informative stimulus to present next. A Bayesian variant of these procedures, the QUEST procedure (Watson & Pelli, 1983), has become popular over the past few decades. This procedure introduced the idea of a prior probability density for the psychometric function parameters. It assumes a single stimulus dimension, sampled periodically in the log domain, and two possible trial outcomes (e.g., “yes” and “no”). It also assumes a constant slope of the psychometric function over the course of the experiment and therefore estimates only the threshold parameter.

Several years later, refinements of Bayesian adaptive procedures were proposed to offer the possibility of simultaneously estimating more than one parameter (i.e., including slope) of the psychometric function (King-Smith & Rose, 1997; Kontsevich & Tyler, 1999; Snoeren & Puts, 1997). In its recent iteration, called QUEST+, Watson (2017) extended the work of Sims and Pelli (1987) and Kontsevich and Tyler (1999), and adopted a stimulus placement rule that minimizes the expected entropy of the posterior probability distribution of the two parameters. In addition, QUEST+ allows “more than two trial outcomes, an arbitrary relationship between stimulus and psychometric function parameters, [and] an arbitrary sampling of stimulus and parameter dimensions” (Watson, 2017, p. 1).

Surprisingly, QUEST+ is barely used despite several publicly available codes (written for MATLAB by Brainard, 2017; or Jones, 2018; or for Mathematica by Watson, 2017) aimed at facilitating its successful implementation. We found fewer than 10 published peer-reviewed empirical studies reporting QUEST+ results: some evaluated how perceived color and material combine in object selection (Radonjić et al., 2019), some assessed size perception (Linton, 2021) or visual acuity (Ng et al., 2021), one compared perceptual thresholds of the perceived quality of computer-generated images measured in the laboratory or online (Myrodia et al., 2021), and finally, three estimated contrast sensitivity functions (Dekker et al., 2020; Elfadaly et al., 2020; Farahbakhsh et al., 2019). To our knowledge, the study by Farahbakhsh et al. (2019) is the only one comparing the empirical performance of QUEST+ with another psychophysical measurement method, the staircase procedure. Overall, the authors found good agreement between the two methods, and emphasized the greater test-retest reliability and the rapidity of testing of the QUEST+ procedure. Such empirical tests are of great importance in assessing the efficacy of psychophysical methods in human observers. Indeed, in computer simulations, the chosen observer model might not perfectly reflect a real observer’s behavior (Alcalá-Quintana & García-Pérez, 2007; García-Pérez & Alcalá-Quintana, 2009; Madigan & Williams, 1987). Human observers are indeed subject to failure due to fatigue, boredom, or attentional fluctuations for instance.

In the present study, we extended the empirical comparison between the QUEST+ and other psychophysical procedures initiated by Farahbakhsh et al. (2019) in several important ways (besides methodological differences that will be detailed later). First, we added the CS procedure (considered to yield accurate parameter estimates, but at the cost of a large number of trials) to the comparison. Second, and importantly, we compared estimation precision not only for threshold, but also for slope parameters. Third, we assessed whether the use of the QUEST+ procedure could be generalized to three different stimulus dimensions involved in perception—size (Experiment 1), orientation (Experiment 2), and temporality (Experiment 3)—and under increasing task demands (foveal vs. peripheral stimuli discrimination and low- vs. high-level dimensions).

In general, we expected to find comparable threshold and slope estimates between procedures. In line with Farahbakhsh et al. (2019), we should indeed observe good correlations for thresholds. Regarding slope parameters, sensitivity estimates in the QUEST+ procedure also should not differ from the estimates obtained with the CS procedure. This hypothesis is based on Snoeren and Puts (1997), who obtained accurate threshold and slope parameters after 64 trials in their Bayesian adaptive procedure simulations, even though other simulations needed at least 200 trials to obtain accurate slope estimates (Kontsevich & Tyler, 1999). One of the caveats on adaptive procedures, according to Kaernbach, 2001, is the serial dependency of the obtained data that reduces slope estimation accuracy. This issue should however be overcome in parametric procedures such as QUEST+ which use a multidimensional array of parameters to determine trial placements (Kaernbach, 2001; Watson, 2017). For the staircase procedure, initially designed to measure thresholds only, we expected lower correlations for slope estimates with the other two procedures. To estimate slope, we need to develop psychometric functions from trial-by-trial data. To reduce serial data dependency and test a larger range of stimulus values in the staircase procedure (Cornsweet, 1962), we randomly interleaved three independent tracks with three different and widely spaced initial stimulus values. We then fitted psychometric functions, recalculated thresholds, and estimated slopes. Yet, this process does not guarantee an optimal reconstruction of the underlying psychometric function and therefore an accurate slope estimation. In the literature, slopes obtained from adaptive procedure data were sometimes too steep (Kaernbach, 2001; Leek et al., 1992; Treutwein & Strasburger, 1999).

Finally, we also examined whether fast learning occurred through the course of our 1-hour experiments. A variety of factors are known to affect learning in perceptual tasks, such as the nature and the number of perceptual dimensions relevant to the task, external noise, familiarity, task complexity, error feedback, and attention (see Dosher & Lu, 2017; Fahle, 2002; Fine & Jacobs, 2002; and Maniglia & Seitz, 2018; for reviews). As shown by Hawkey et al. (2004), an early and rapid performance improvement could arise from both procedural learning (i.e., learning the response to the task demands) and perceptual learning (i.e., an increase in the precision with which stimuli are represented, stored, or compared). We did not expect perceptual learning to occur when visual discrimination was made for single low-level dimensions in the fovea (like object size in the first experiment), because this task should be easy for participants. In contrast, we could not rule out a potential learning effect in our last two experiments where discrimination was made in peripheral vision and on higher-level dimensions. Procedural learning, for its part, could occur through trials in any of our experiments, and particularly when the task demand is important (such as in temporal judgments). If learning, procedural and/or perceptual, takes place, psychometric function parameters could be impacted, which could interfere with the primary goal of our study to compare psychophysical measurement methods.

## Methods

### General methods

#### Participants

Participants were recruited through the online platform of the University Paris Cité. Participants either obtained course credit or received compensation of €10 per hour. All participants were naive about the purpose of the study, except for the three authors, who also performed at least two of the experiments. Participants all had normal or corrected-to-normal vision. They gave their informed written consent prior to the experiment. Experiments were conducted in accordance with the principles of the Declaration of Helsinki and approved by the local ethics committee (00012021-118-PAEYE).

#### General procedure and design

Participants performed a size (Experiment 1), orientation (Experiment 2), or temporal (Experiment 3) perception task. Power analysis showed that 20 participants were needed to obtain the same correlation between psychometric function parameters (*r* = .60) as that in Farahbakhsh et al. (2019) with power of .90. In each experiment, psychophysical measurements were made in a yes/no discrimination task (according to Klein’s, 2001 classification), using three different procedures: CS, staircase, and QUEST+ (Q+). The procedure order was counterbalanced across participants (six different orders were randomly assigned).

Basically, one stimulus (or two stimuli in Experiment 3) that varied from trial to trial along one physical dimension (size, orientation, or onset asynchrony) was briefly presented on a gray screen. The stimulus was displayed in either central (Experiment 1) or peripheral (Experiments 2 and 3) vision, and had to be compared on the specific tested physical dimension to a subsequent reference stimulus which was, again, presented in either central or peripheral vision. Stimuli were generated using the Psychophysics Toolbox extensions for MATLAB (Brainard, 1997; Kleiner et al., 2007; Pelli, 1997). The specificity values for each experiment are further detailed in dedicated sections.

The CS procedure, considered as the gold standard of psychophysical measurement methods, consisted of a unique block of 180 to 184 trials where nine stimulus values were randomly presented. We presented a large number of trials to guarantee precise estimations of the psychometric function parameters. Stimulus values were uniformly distributed over a predetermined range of values that encompassed the participants’ supposed 50% threshold. In each experiment, this threshold was either taken from our previous study (Pressigout et al., 2020 for Experiment 1) or determined based on pilot participants’ performance (these participants were not included in the study).

For the staircase procedure, three independent threshold tracks were randomly interleaved. They were simple up-down tracks (i.e., the stimulus value changed by a fixed step size after each response) that began at stimulus levels far above, far below, or at the supposed threshold (see the detailed procedures below for the specific initial values). Each track was stopped after 15 response reversals or 10 presentations of the maximum or minimum stimulus value. The whole staircase procedure could continue up to 150 trials. At the end of the procedure, a mean threshold value was classically computed based on the last five reversals for each track. Then, the 50% threshold estimation was computed as the mean of the three tracks’ threshold mean values. By developing psychometric functions, we were able to obtain second threshold estimations, as well as slope estimations.

The QUEST+ procedure was programmed using a free extension for MATLAB, made available in 2017 by Brainard on his GitHub repository, which takes up Watson’s implementation on Mathematica in his 2017 article. The underlying function was a cumulative normal distribution. Such a model describes the probability of responding “yes” (*P*_yes_) as a function of the stimulus level (*x*). A “yes” response is expected for the highest stimulus values, and its probability increases with the increase in stimulus values. This function has been implemented in Brainard’s code as follows:

1 $${P}_{yes}= lapse+\left(1-2\ast lapse\right)\ast 0.5\ast \left(1+\mathit{\operatorname{erf}}\left(\frac{x-m}{sd\times \sqrt{2}}\right)\right)$$

with *m* and *sd* corresponding to the mean and standard deviation of the fitted normal distribution and *lapse* to the lapse rate (the probability of an incorrect response which is independent of stimulus value). The standard MATLAB function *erf*(z) returns the error function evaluated for each element of *z*. This MATLAB implementation is equivalent to the function proposed for Mathematica by Watson (2017, p. 9).

The domain of the stimulus values and that of the psychometric function parameters were obtained from our previous data, either from Pressigout et al.'s study (2020) or from our pilot experiments. We decided to use uniform distributions of stimulus and parameter domains (i.e., all values were equally likely), meaning that we did not introduce any explicit Bayesian priors—although we made assumptions about the models and the domain boundaries. This way, we avoided the additional risk of measurement biases or experimenter errors (see Farahbakhsh et al., 2019). Because the challenge of adaptive psychophysical procedures is to maximize efficiency and minimize experiment duration (Leek, 2001; Treutwein, 1995), we chose to stop the QUEST+ procedure after 64 trials, based on Snoeren and Puts’s simulations (1997).

In each experiment, participants all started with a training phase of 64 trials, for which stimulus values were randomly drawn from the stimulus value domain of the QUEST+. These data were not analyzed. The overall test duration was around 45 minutes to 1 hour. When eye fixations were controlled, each block of trials started with a five-point calibration procedure.

### Data analyses

For the three psychometric methods, we fitted a cumulative Gaussian function to our data using the MATLAB *fminsearch* algorithm. Note that in the staircase procedure, thanks to the three interleaved adaptive tracks, we sampled comparable distributions of stimulus values as in the CS procedure, and obtained the same sample sizes by constituting bins of stimulus values. It is also important to note that threshold values obtained with such fits were highly correlated with threshold estimates obtained at the end of the staircase procedure with the conventional method, *r* range: .86–.99, all *p*_s_ < .001. These new estimates were used in our analyses.

Psychometric function parameters were estimated using the maximum likelihood method and were used to define points of subjective equivalence (PSEs) and just-noticeable differences (JNDs). The PSE is the point at which a comparison stimulus is judged equal to a reference stimulus (i.e., both positive or negative responses are equally likely to be made), and gives information about possible response biases (the degree to which “yes” or “no” responses are preferred) or systematic errors. The JND is the smallest detectable difference between two stimuli, and was calculated as follows: *JND* = (*x*_.75_ − *x*_.25_)/2, with *x*_.75_ and *x*_.25_ corresponding to the stimulus values at which the probability of a given response (e.g., “yes”) is equal to 75% and 25%, respectively. The JND is a measure of the spread of the psychometric function and thus is inversely related to the slope. Because JND distributions were moderately asymmetric and skewed, we transformed our JND data for analyses. We chose a logarithmic transformation based on the Box–Cox procedure (Box & Cox, 1964).

For the main analyses, linear mixed models (LMM; R package *lme4*, Bates et al., 2015) were used to examine a potential effect of the different psychophysical procedures (QUEST+, CS, and staircase) on PSE and JND estimates. As random effects, we had intercepts for participants. For *F* tests on fixed effects, we used Satterthwaite’s approximation (*lmerTest package*, Kuznetsova et al., 2017). Marginal *R*^2^ values are reported as a measure of the variance explained by fixed factors (Nakagawa & Schielzeth, 2013). In order to corroborate absence of effects, we also conducted Bayesian repeated-measures analyses of variance (ANOVAs) (JASP Team, 2022, v.0.16.1). In these analyses, we compared the null model that contains only the grand mean with the model that contains the main effect of the procedure (or block, see below). Results are expressed as Bayes factors (*BF*_01_) for each model against the null model (the prior values were set to the JASP default values).

To quantify the degree to which the three procedures are related, we calculated Pearson or Spearman pairwise correlations (depending on the normality of distributions). Because correlations only measure the strength of a linear relationship between two variables, we conducted Bland–Altman analyses (Bland & Altman, 1986) to further assess the agreement between procedures. These analyses quantify the agreement between two quantitative measurements by studying their mean difference and constructing limits of agreement (corresponding to ±1.96 × *SD* of the differences).

Finally, we evaluated the potential impact of learning on psychometric functions. We first created a “block” factor that corresponds to the trials for the first, second, and third implemented procedures, independently of the method used. Indeed, the order in which the specific procedures were implemented was counterbalanced, as previously mentioned in the general procedure and design section. Then, we performed linear mixed effect analyses of the relationship between PSEs or JNDs and blocks of trials (fixed effects). As random effects we had intercepts for participants. To examine if a response bias existed, we also compared mean PSE values obtained for each block (or procedure) to the value of the reference stimulus, using Bayesian one-sample *t*-tests.

## Experiment 1

The first experiment aimed at comparing PSEs and JNDs obtained with the three procedures in a typical psychophysical task: the size judgment of a disk presented in central vision.

### Participants

Twenty-two participants (19 females) aged 18 to 41 years (*M* = 23.8; *SD* = 6.5) contributed data in this experiment (three of them were the authors). An additional four adults were tested but excluded because of failure to complete the experiment (1) or because the standard deviation of the psychometric function estimated by the QUEST+ algorithm reached the ceiling (3). It is thus likely that their JNDs do not reflect their true perceptual sensitivity. In addition, for two of these participants, the adaptive tracks of the staircase procedure did not converge.

### Stimuli and material

Participants were installed in a dimly lit room seated about 60 cm from a 19-inch Dell laptop monitor (resolution 1080 × 1920; 60 Hz refresh rate).

Stimuli were black disks presented on a 4 cd/m^2^ luminance gray background. Disk contours were homogeneously blurred by a cumulative Gaussian gradient covering 20% of the diameter (see Pressigout et al., 2020). The reference disk had a constant diameter of 1.6 degrees of visual angle (dva) whereas the diameter of the test disks could vary from 1.2 to 2 dva. A 0.7 × 0.7 dva black cross (line width of 2 pixels) served as a fixation point. For each participant, a different visual noise mask was generated at the beginning of each psychometric procedure. The masks consisted of 15 × 15 dva squares composed of gray-level pixels whose values were randomly picked between 0 and 100 (0 being black and 255 white).

### Procedure and design

**Fig. 1:**
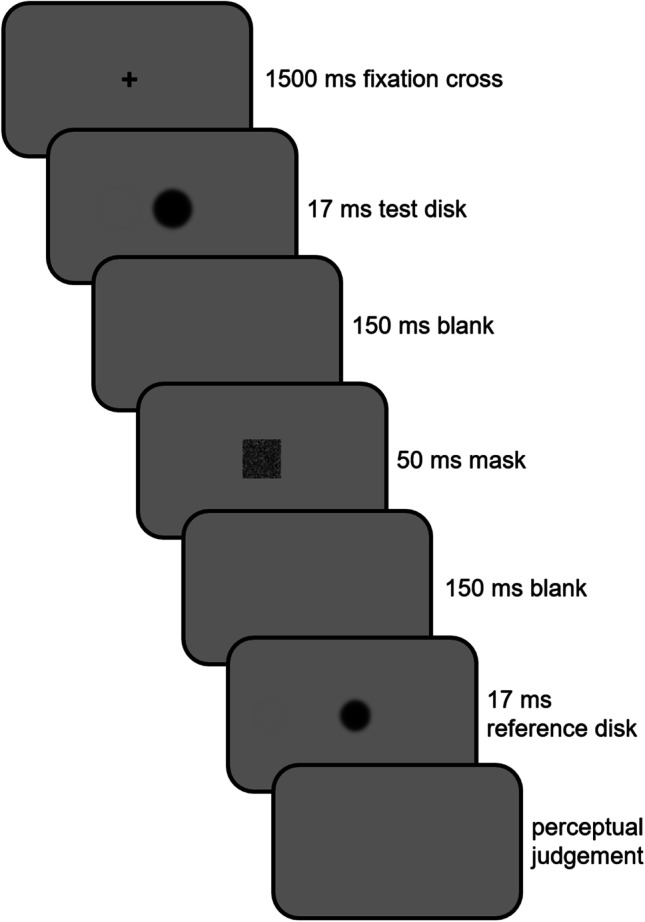
Trial structure in Experiment 1. The stimuli are not drawn to scale

A trial always started with the presentation of a central fixation cross (Fig. 1, frame 1). After 1500 ms, the cross was replaced for one frame (i.e., 17 ms, Fig. 1, frame 2) by a test disk that could vary in size. After a 150-ms blank screen, a noise mask was presented for 50 ms to avoid retinal persistence, and immediately followed by another 150-ms blank screen (Fig. 1, frames 3 to 5). Finally, the reference disk was displayed at the center of the screen for the same duration as the test disk (Fig. 1, frame 6). Participants had then to indicate whether they perceived the first disk as being smaller or larger than the reference disk by pressing the down- or up-arrow keys, respectively.

In the CS procedure, the test disk diameter domain consisted of seven values, from 1.3 to 1.9 dva, spaced by 0.1 dva. Each diameter value was randomly presented 24 times during the session. Two test disks of more extreme diameters, 1.2 and 2 dva, were also inserted eight times each throughout the course of the session.

**Fig. 2:**
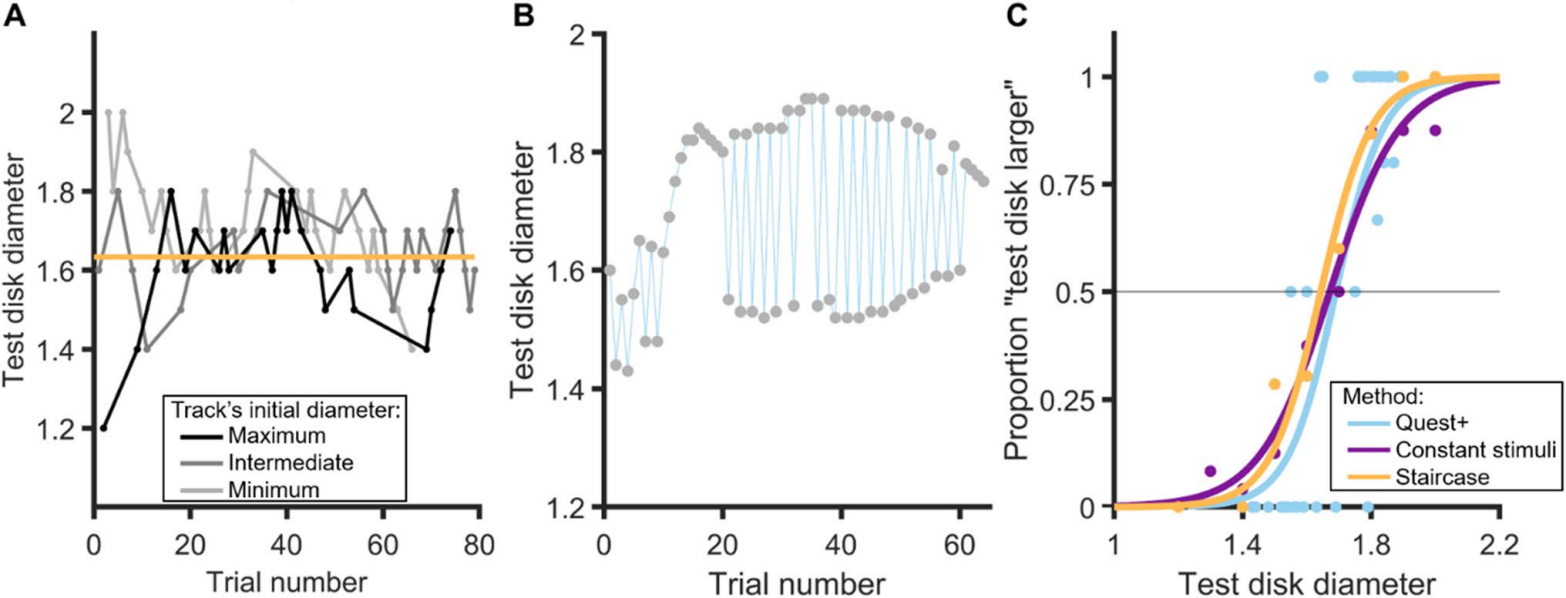
Data for one representative participant in Experiment 1. **A** Interleaved simple up-down tracks in the adaptive staircase procedure. The horizontal orange line indicates the 50% threshold (1.63 here) obtained by averaging the thresholds (i.e., means of the last five reversals) of each track. **B** Trials presented in the QUEST+ procedure. **C** Psychometric functions for each procedure. PSE values obtained from the fitted data were 1.69, 1.67, and 1.64 for the QUEST+, CS, and staircase procedures, respectively. The corresponding JND values were .08, .12, and .09

In the staircase procedure, the initial values of the stimuli were 1.2, 1.6, and 2 dva, depending on the simple up-down track considered. In each track, the stimulus diameter changed by 0.2 dva after the first two response reversals and by 0.1 dva for the following reversals. Figure 2A illustrates the time course of the staircase procedure for one participant.

In the QUEST+ procedure, the test disk diameter domain consisted of all possible values from 1.2 to 2 dva, linearly spaced by 0.01 dva. The parameter domain was trivariate. The possible values for the mean were the same as for the stimulus domain. Standard deviation could take values between 0.02 and 0.3 (0.01 dva step), and lapse values between 0 and 0.06 (0.01 dva step). Fig. 2B shows the time course of the QUEST+ procedure for the same participant as in Fig. 2A. Figure 2C illustrates the psychometric functions obtained for each procedure in one representative participant. The PSEs and JNDs used in the analyses below were obtained from these functions.

### Results

Because outliers within data sets can distort the correlation coefficient value (Goodwin & Leech, 2006)*,* we used the *Sn* method (Rousseeuw & Croux, 1993) with a criterion of 3, as recommended by Jones (2019) for psychophysical data, to detect possible PSE and JND outliers before the analyses. At the end, only one participant was removed as an outlier (note that when data included this participant, statistical conclusions did not change; see supplementary Tables S1 and S2).

#### Number of trials

Parameter estimations were based on 64 valid trials in the QUEST+ procedure, 184 trials in the CS procedure, and 76 ±8 trials on average in the staircase procedure (range of participants’ contribution: 61–95 trials).

#### PSEs

**Fig. 3:**
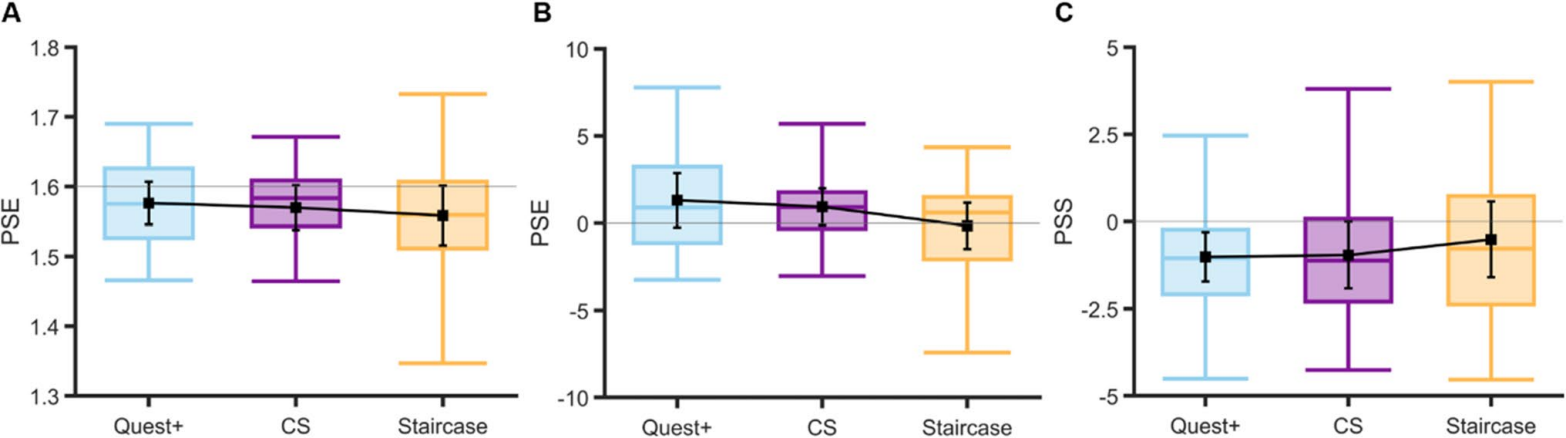
Boxplots of PSEs obtained for each psychometric procedure (fences are located at 2 × IQR above the 75th percentile and below the 25th percentile) **A** in the 21 participants of Experiment 1 and **B** in the 20 participants of Experiment 2. **C** PSSs obtained in the 21 participants of Experiment 3. Black squares: mean PSEs or PSSs; error bars: 95% confidence intervals

Figure 3A shows the boxplots of PSEs for each procedure. PSE values did not change across procedures (*M*_Q+_ *=* 1.58 dva*, SD*_Q+_ *=* 0.07, *range*_Q+_: 1.47–1.69; *M*_CS_ = 1.57 dva*, SD*_CS_ = 0.07, *range*_CS_: 1.36–1.67; *M*_Staircaise_ = 1.56 dva, *SD*_Staircase_ = 0.09, *range*_Staircase_: 1.41–1.73), LMM: *F*(2,42) = 1.46, *p* = .24, and *BF*_01_ = 2.8. A Bayesian one-sample *t*-test revealed anecdotal evidence for bias when PSEs were measured using the CS, *BF*_01_ = 0.87, and staircase procedures, *BF*_01_ = 0.58, but not for the QUEST+ procedure, *BF*_01_ = 1.4.

Pairwise correlations between PSE values were significant, all *r*(19) ≥ .79 and all *p*s < .001. The strongest correlation, *r*(19) = .86, *p* < .001, was observed for the two adaptive procedures (see Table 1).

**Table 1:** Pairwise Pearson (*r*) or Spearman (*r*_*s*_) correlations between PSE values and between JND values of the different procedures, for each experiment. *P*-values less than .05 shown in bold

		Expt 1		Expt 2		Expt 3	
		*r*	*p*	*r*	*p*	*r*	*p*
PSE (PSS)	Q+ and CS	***r***_***s***_**(19) = .85**	**<.001**	***r*(18) = .69**	**<.001**	*r*(19) = .25	.27
	Q+ and staircase	***r***_***s***_**(19) = .86**	**<.001**	***r*(18) = .45**	**.046**	*r*(19) = .42	.057
	CS and staircase	***r***_***s***_**(19) = .79**	**<.001**	***r*(18) = .45**	**.045**	***r*(19) = .46**	**.036**
JND	Q+ and CS	***r*(19) = .75**	**<.001**	***r***_***s***_**(18) = .62**	**.005**	*r*(19) = .40	.073
	Q+ and staircase	*r*(19) = .32	.16	*r*_*s*_(18) = .42	.065	*r*(19) = −.14	.055
	CS and staircase	*r*(19) = .19	.41	***r***_***s***_**(18) = .59**	.**007**	***r*(19) = .46**	**.035**

**Fig. 4:**
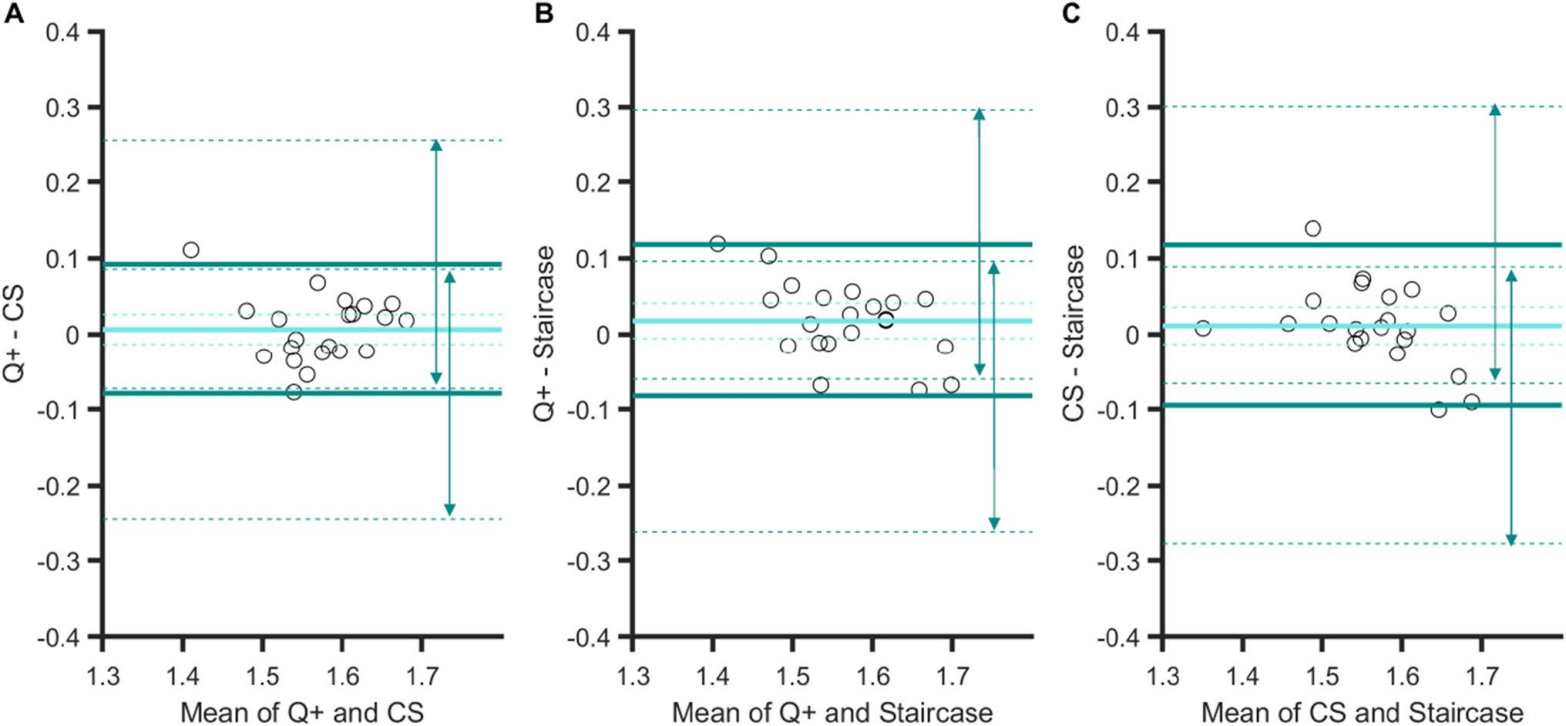
Bland–Altman plots for PSEs in Experiment 1. Agreements between: **A** the QUEST+ and CS procedures, **B** the QUEST+ and staircase procedures, and **C** the CS and staircase procedures. Light blue line: mean difference in PSE between two specific procedures (showing a possible bias for one procedure over another). Dark green lines: upper and lower limits of agreement (i.e., 1.96 × *SD* from the mean) between procedures. Dashed lines indicate 95% confidence intervals. For the sake of clarity, the extent of confidence interval boundaries is represented by arrows

These strong correlations between PSEs are confirmed by the Bland–Altman plots in Fig. 4. Indeed, the 95% confidence intervals (CI) of the limits of agreement (dark green arrows) all overlap, and the spreads of the agreement intervals are very similar (range: 0.17–0.21 dva). Finally, there was no significant bias for one procedure over another (zero values are within the 95% CIs of the mean difference, light blue dashed lines in panels A to C).

#### JNDs

**Fig. 5:**
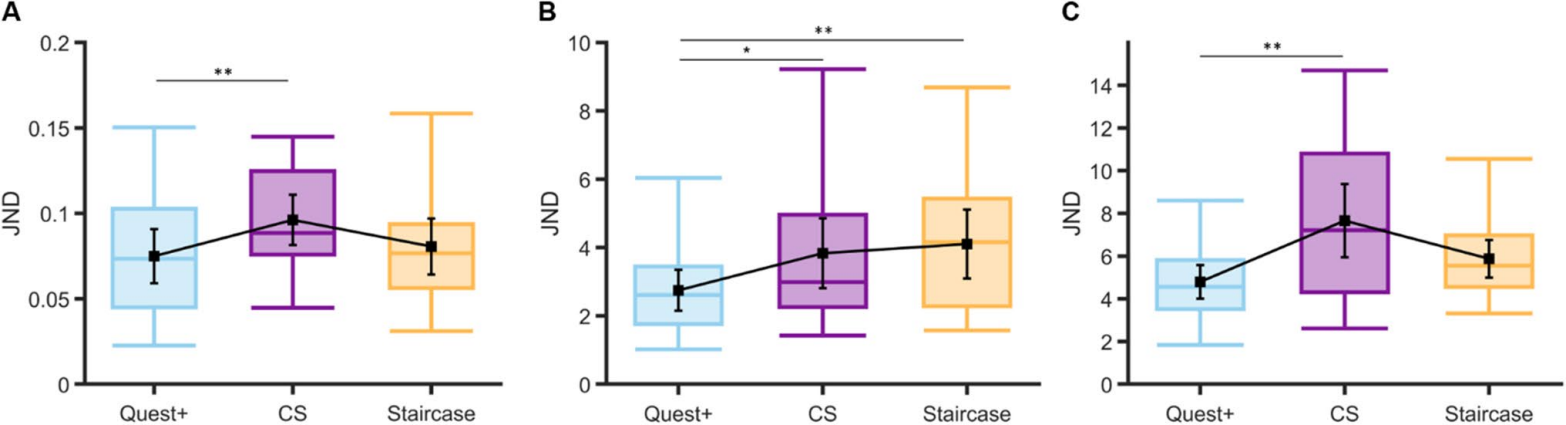
Boxplots of JNDs obtained for each psychometric procedure (fences are located at 2 × IQR above the 75th percentile and below the 25th percentile) **A** in the 21 participants of Experiment 1, **B** in the 20 participants of Experiment 2, **C** in the 21 participants of Experiment 3. Black squares: mean JNDs, error bars: 95% confidence intervals. * *p* < .05 and ** *p* < .01 for Tukey *HSD* post hoc comparisons

JNDs were slightly affected by the procedure, as illustrated in Fig. 5A, LMM, *F*(2,42) = 5.45, *p* = .008; marginal *R*^2^ = .083. Tukey’s honestly significant difference (*HSD*) post hoc analyses (*emmeans* package, Lenth, 2020) showed that only mean JNDs were smaller for the QUEST+ procedure (*M*_Q+_ = .075, *SD*_Q+_ = .035, *range*_Q+_: −.02 to .17) in comparison with the CS procedure (*M*_CS_ = .096, *SD*_CS_ = .032, *range*_CS_: .05**–**.17), *p* = .008. QUEST+ and CS did not differ from the staircase procedure (*M*_Staircase_ = .081, *SD*_Staircase_ *=* .036, *range*_Staircase_: −.03 to .21), *p* = .59 and *p* = .088, respectively.

**Fig. 6:**
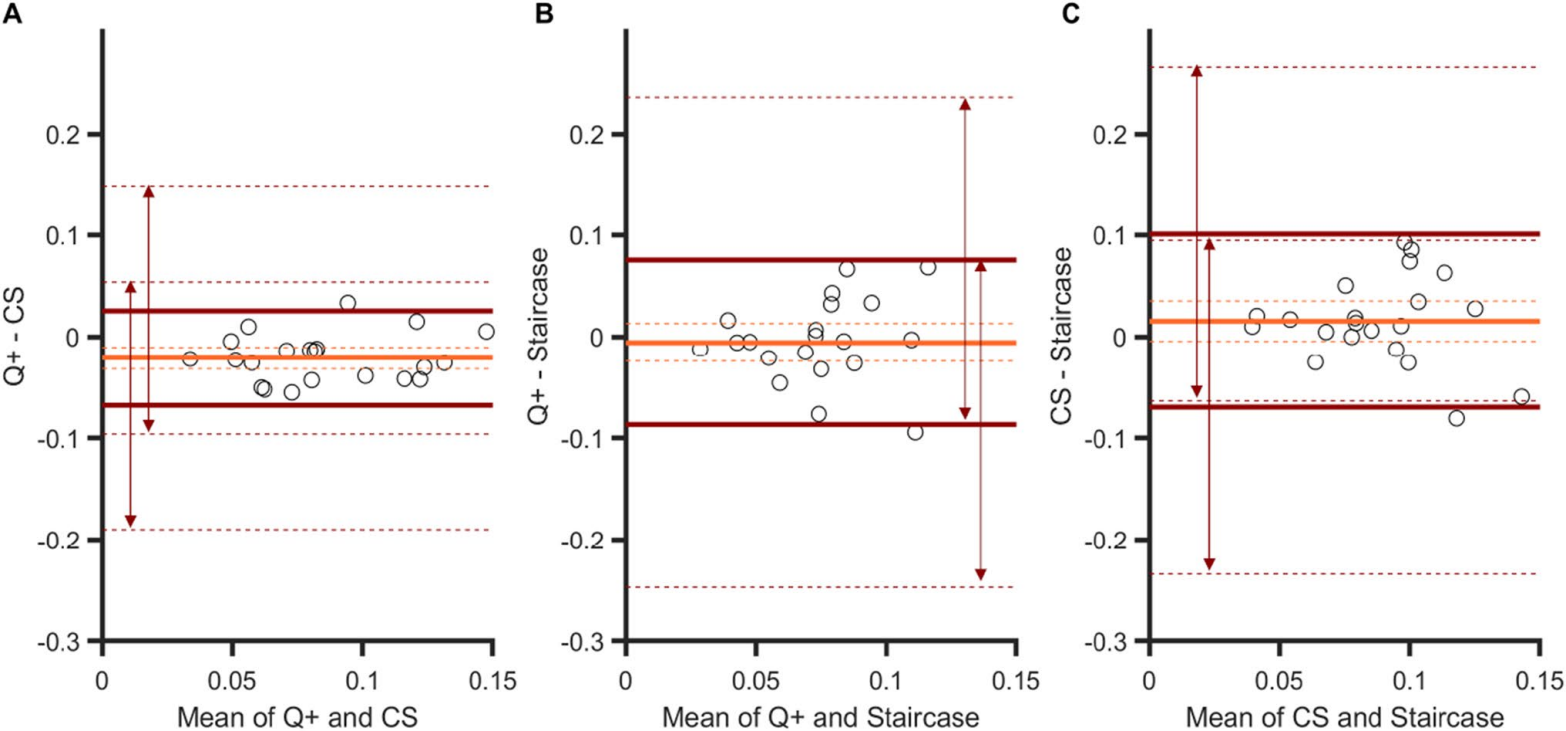
Bland–Altman plots for JNDs in Experiment 1. Agreements between: **A** the QUEST+ and CS procedures, **B** the QUEST+ and staircase procedures, and **C** the CS and staircase procedures. Orange line: mean difference in JNDs between two specific procedures (showing a possible bias for one procedure over another). Brown lines: upper and lower limits of agreement (i.e., 1.96 × *SD* from the mean) between procedures. Dashed lines indicate 95% confidence intervals. For the sake of clarity, the extent of confidence intervals’ boundaries is represented by arrows

The only significant correlation between JNDs was observed for the QUEST+ and CS procedures, *r*(19) = .75, *p <* .001 (*p* values were .16 and .41 for the other two correlations, see Table 1). This is consistent with the Bland–Altman plots (Fig. 6) that show the strongest agreement between the QUEST+ and CS procedures, with an interval range of 0.10 (vs. 0.17 for the other two comparisons). In general, however, agreement between procedures was good; their 95% CIs of agreement limits indeed overlap (see arrows in all panels). Importantly, for the QUEST+ procedure, JNDs were significantly smaller than JNDs measured in the CS procedure (the zero value is outside the 95% CI of the mean difference, panel A), whereas no such biases were observed for the other procedure comparisons.

#### Learning effect

**Fig. 7:**
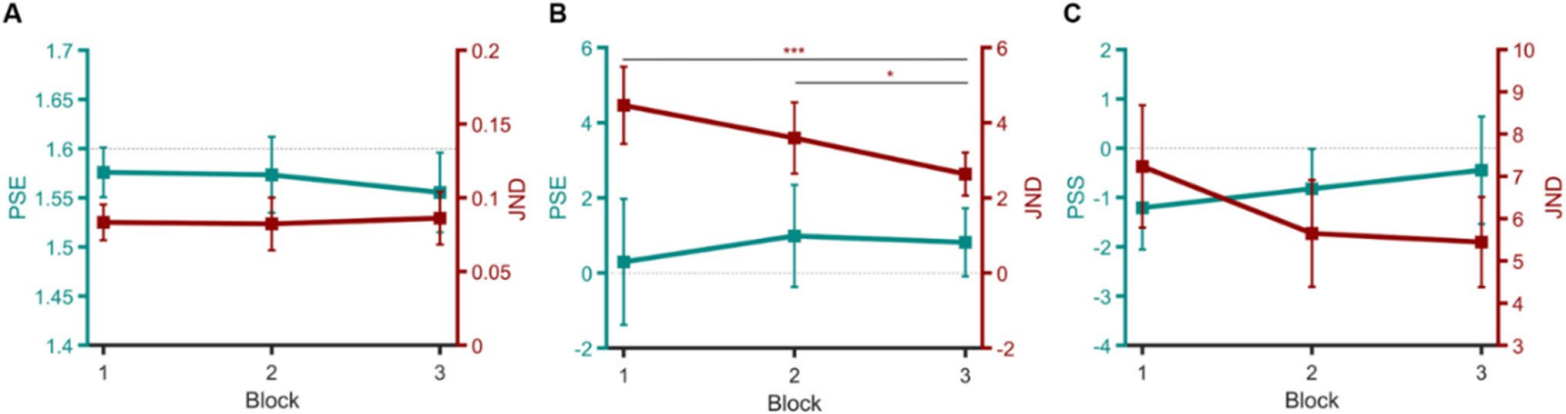
Mean PSEs and JNDs as a function of trials, obtained **A** in the 21 participants of Experiment 1, **B** in the 20 participants of Experiment 2, **C** in the 21 participants of Experiment 3. Error bars: 95% confidence intervals. * *p* < .05; *** *p* < .001 for Tukey *HSD* post hoc comparisons

To examine whether there was any learning effect, we compared the mean values of PSEs and JNDs across the first, second, and third blocks of trials (independently of the procedure). We did not observe any block effect on PSEs, LMM: *F*(2,42) = 2.30, *p* =.11, and *BF*_01_ = 1.62, nor on JNDs, LMM: *F*(2,42) = 0.28, *p* =.76, and *BF*_01_ = 6.34; they remained constant through the entire experiment (see Fig. 7A).

## Experiment 2

In this experiment we compared PSE and JND estimates obtained from the QUEST+, CS, and staircase procedures for feature orientation judgments, another low-level physical dimension. We increased task difficulty by presenting stimuli in peripheral vision.

### Participants

Twenty-three participants (18 females) aged 18 to 42 years (*M* = 22.7, *SD* = 6.2) contributed data in this experiment (three of them were the authors). An additional 11 participants were tested but excluded for failure to calibrate (1), because the final standard deviation estimated by the QUEST+ algorithm reached the maximum ceiling (3), because they did not perform the task correctly, yielding aberrant values for at least one procedure (4), or for a combination of the two latter points (3).

### Stimuli and material

In this experiment, as in the subsequent one, stimuli were projected on a screen with dimensions of 598 × 344 inches (1280 × 720 pixels) by a digital micromirror video projector (PROPixx Full, VPixx Technologies, 244 Hz). The room was lit only by the projected image. Participants sat ~140 cm from the screen, with their head stabilized by a chin and forehead rest. We used a video-based eye tracker (EyeLink 1000 Plus; SR Research®) to control for central fixation during peripheral stimulus presentation.

Stimuli, two 2 × 2 dva black and white gabors (Gaussian enveloped sinusoidal gratings, with spatial frequency = 1.3 cycles per degree, contrast = 100%, spatial constant of the Gaussian hull function = 0.68 dva, and phase = 1), were displayed on a 7.5 cd/m^2^ luminance gray background. The reference gabor was tilted 20° to the right whereas the test gabor tilt angles could vary from 5° to 35° to the right. A 0.7 × 0.7 dva black cross (2 pixels line width) served as a fixation point. At the beginning of each psychometric procedure, and for each participant, a different visual noise mask was generated. The masks consisted of 5 × 5 dva squares composed of gray-level pixels whose values were randomly picked between 0 and 100 (0 being black and 255 white).

### Procedure and design

**Fig. 8:**
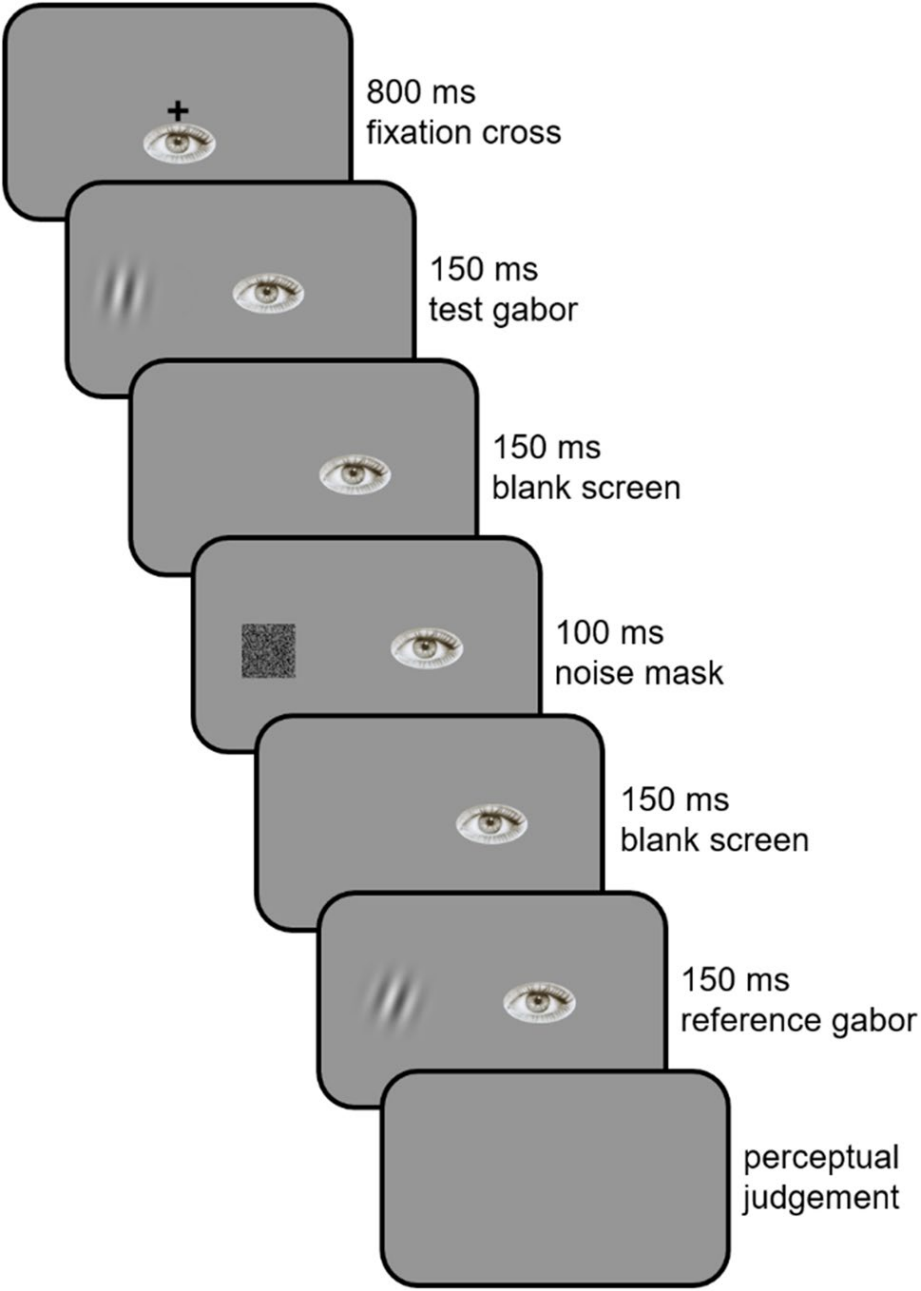
Trial structure in Experiment 2. Stimuli are not drawn to scale

A trial always started with the presentation of a fixation cross displayed at the center of the screen (Fig. 8, frame 1). After 800 ms, the test gabor appeared at 14 dva eccentricity to the left of the fixation cross, which simultaneously disappeared (Fig. 8, frame 2). Participants were still instructed to fixate its location for the entire trial. The test gabor remained on the screen for 150 ms. After a 150-ms blank screen (Fig. 8, frame 3), it was replaced by a 100-ms noise mask (Fig. 8, frame 4). After another 150-ms blank screen (Fig. 8, frame 5), the reference gabor was displayed at the exact same location as the test gabor and for the same duration (Fig. 8, frame 6). Participants had then to indicate whether they perceived the test gabor as being more tilted to the left or to the right than the reference gabor by pressing the left- or right-arrow keys, respectively (Fig. 8, frame 7). During the whole trial, we ensured that participants kept fixating on a 1.5-dva-radius area around the center of the screen (where the fixation cross first appeared). If a saccade or a drift outside this area was detected, the trial was aborted and played again later on (in the CS procedure) or immediately (in the adaptive procedures).

In the CS procedure, the set of tilt angle differences between the test and reference gabors consisted of seven possible values from −9° to 9° (a positive value corresponding to a clockwise rotation) spaced by 3°. Each tilt angle difference was randomly presented 24 times during the session. Two ±15° tilt angle differences were also inserted eight times each throughout the course of the session.

In the staircase procedure, the initial tilt angle differences were −28°, 0°, and 28°, depending on the simple up-down track considered. Then the boundaries were set to ±24°. In each track, the angle difference changed by 4° after the first two response reversals and by 2° for the following reversals.

In the QUEST+ procedure, the set of tilt angle differences consisted of all the possible values from −24° to 24° linearly spaced by 2°. The possible values for the mean were the same as for the stimulus domain. Standard deviation could take values between 1 and 20 (step of 0.1), and lapse, values between 0 and 0.08 (step of 0.01°).

### Results

Three outliers were removed from the analyses (based on the *Sn* method, with a criterion of 3). An additional analysis with all participants showed that the exclusion of these outliers did not change the statistical conclusions on PSEs and JNDs, but that some correlations between estimates were artificially increased (see supplementary Tables S1 and S2).

#### Number of trials

Parameter estimations were based on 64 valid trials in the QUEST+ procedure, 184 trials in the CS procedure, and 108 trials on average in the staircase procedure (range of participants’ contribution: 82–150 trials).

#### PSEs

As illustrated in Fig. 3B, the effect of procedure on PSEs did not reach significance, *F*(2,40) = 3.08, *p* =.057, even if evidence speaking in favor of this null hypothesis is anecdotal, *BF*_01_ = 1.23. For the QUEST+, CS, and staircase procedures, the mean PSEs were 1.31° (*SD*_Q+_= 3.32, *range*_Q+_: −7.21 to 10.2), 0.94° (*SD*_CS_ = 2.25, *range*_CS_: −6.08 to 13.02) and −0.15° (*SD*_Staircase_ = 2.83, *range*_Staircase_: −5 to 12.44), respectively. Participants were accurate in all procedures, as the mean PSE values never differed from the critical value of zero (Bayesian one-sample *t*-tests, *BF*_01_ range: 1.02–4.19, and see the 95% CIs in Fig. 3B).

The highest correlation between the PSE values was observed for the QUEST+ and the CS procedures, *r*(18) = .69, *p <* .001. The other two coefficients were similar although slightly smaller, both *r*(18) = .45, *p =* .045 and .046 (see Table 1).

**Fig. 9:**
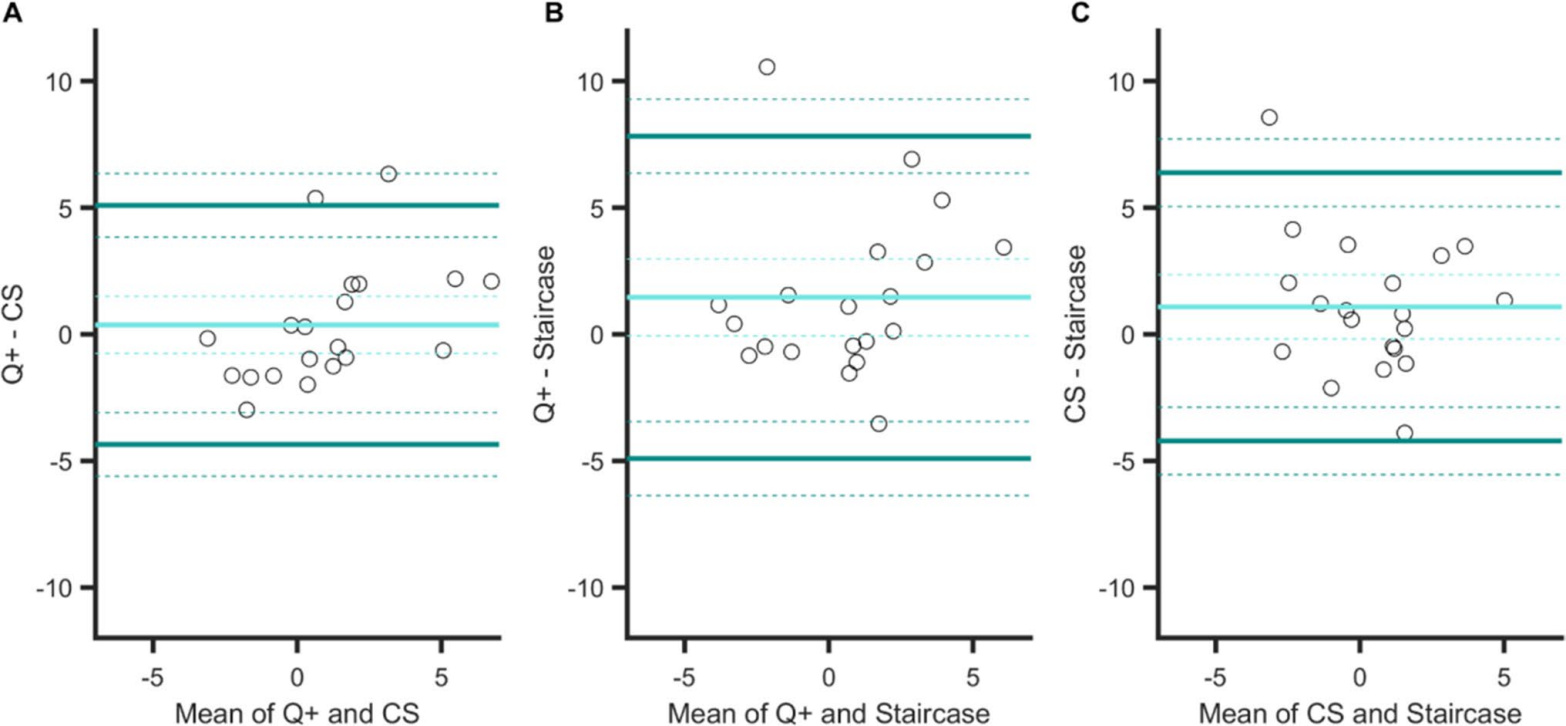
Bland–Altman plots for PSEs in Experiment 2. Agreements between: **A **the QUEST+ and CS procedures, **B** the QUEST+ and staircase procedures, and **C** the CS and staircase procedures. Light blue line: mean difference between two specific procedures (showing a possible bias for one procedure over another). Dark green lines: upper and lower limits of agreement (i.e., 1.96 × *SD* from the mean) between procedures. Dashed lines indicate 95% confidence intervals

Overall, the Bland–Altman analyses on PSEs showed that the agreement between the three procedures was large. Like in Experiment 1, the strongest degree of agreement was obtained between QUEST+ and CS procedures (Fig. 9, panel A): the agreement interval, of 9.4°, was indeed the narrowest [−4.3 to 5.1]. By contrast, the two adaptive procedures showed a smaller agreement, with a larger agreement interval of 12.7° [−4.9 to 7.8] (see panel B). The agreement between the CS and staircase procedures was intermediate, with an agreement interval of 10.6° [−4.2 to 6.4] (see panel C). These analyses confirmed that the procedure had no effect on the PSEs. There was no significant bias for one procedure over another (zero values are within the 95% CIs of the mean difference, light blue dashed lines in panels A to C).

#### JNDs

Contrary to PSEs, the procedure significantly impacted the JNDs (see Fig. 5B), *F*(2,40) = 5.98, *p =* .005, marginal *R*^2^ = .092. Tukey *HSD* post hoc analyses showed that the QUEST+ procedure yielded the highest measures of sensitivity (i.e., the lowest JNDs: *M*_Q+_ = 2.75, *SD*_Q+_ = 1.28, *range*_Q+_: 1.02–6.04) in comparison with both the CS procedure (*M*_CS_ = 3.83, *SD*_CS_ = 2.19, *range*_CS_: 1.43–9.22, *p* = .034) and the staircase procedure (*M*_Staircase_ = 4.10, *SD*_Staircase_ = 2.16, *range*_Staircase_: 1.58–11.84, *p* = .008), which did not differ from each other (*p* = .84).

As for the PSEs, the highest correlation between JNDs was observed for the QUEST+ and the CS procedures, *r*(18) = .62, *p =* .005. Whereas a comparable correlation was obtained for the CS and staircase procedures, *r*(18) = 0.59, *p =* .007, it was lower and failed to reach significance for the two adaptive procedures, *r*(18) = 0.42, *p =* .065 (see Table 1).

**Fig. 10:**
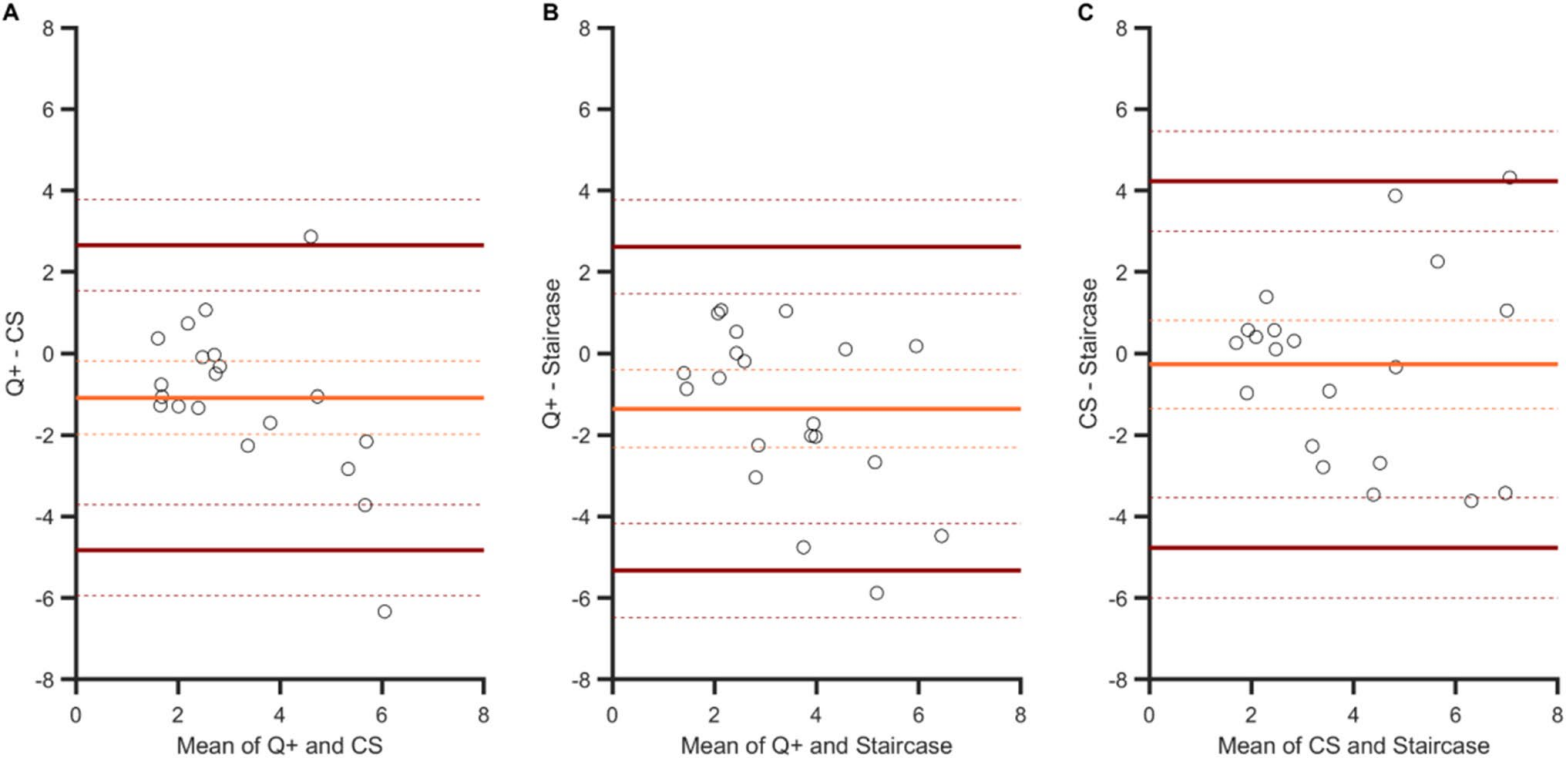
Bland–Altman plots for JNDs in Experiment 2. Agreements between: **A** the QUEST+ and CS procedures, **B** the QUEST+ and staircase procedures, and **C** the CS and staircase procedures. Orange line: mean difference between two specific procedures (showing a possible bias for one procedure over another). Brown lines: upper and lower limits of agreement (i.e., 1.96 × *SD* from the mean) between procedures. Dashed lines indicate 95% confidence intervals

Figure 10 shows the Bland–Altman plots for JNDs. The best agreement was observed for the QUEST+ and CS procedures (agreement interval of 7.5°, [−4.8; 2.7]) and for the QUEST+ and the staircase procedures (agreement interval of 7.9°: [−5.3; 2.6]). The agreement was slightly lower for the CS and staircase procedures (agreement interval of 9°: [−4.2; 4.8]). These analyses also confirmed that the QUEST+ procedure yielded higher sensitivity measures (the zero values are not within the 95% CIs of the mean difference, orange dashed lines in panels A and B) and that CS and staircase procedures measured sensitivity in a similar way (panel C).

#### Learning effect

Figure 7B shows that PSE values remained constant throughout the experiment, which was confirmed by both the LMM, *F*(2,40) = 0.62, *p =* .54, and the Bayesian ANOVA analysis, *BF*_01_ = 4.86.

A learning effect however occurred for sensitivity, LMM: *F*(2,40) = 13.6, *p <* .001; marginal *R*^2^ = 0.16. Tukey *HSD* post hoc analyses showed that JNDs significantly decreased between the first and third blocks (*p <* .001) and the second and third blocks (*p =* .029). A similar trend can be seen between the first and second blocks but failed to reach significance (*p =* .052).

## Experiment 3

In this experiment we compared the two estimates obtained from the QUEST+, CS, and staircase procedures for a higher-level dimension: temporality, assessed for stimuli presented in the periphery. This experiment differed from the preceding ones in that it consisted of the presentation of a single magnitude (in terms of duration) per trial. Participants were asked to perform a temporal-order judgment (TOJ) task, i.e., to report which of two stimuli appeared first. We measured points of subjective simultaneity (PSS), which corresponded to the delay with which one stimulus had to be presented before the other for observers to perceive them as appearing at the same time. The JND represents the smallest interval at which the participants could reliably determine which stimulus was presented first.

### Participants

Twenty-four participants (20 females) aged 18 to 41 years (*M* = 22.9, *SD* = 5.9) contributed data in this experiment (two of them, AP and CP, were the authors). An additional four participants were tested but excluded for failure to perform the task (3) and because they mistakenly pressed the wrong buttons (1).

### Stimuli and material

We used the same material setup as in Experiment 2.

The gray background had a luminance of 20 cd/m^2^. Stimuli were two 1 × 1 dva black and white gabors (spatial frequency = 2 cycles per degree, contrast = 100%, spatial constant of the Gaussian hull function = 0.24° and phase = 1). One gabor was vertical and the other horizontal. A 1 × 1 dva black cross (2 pixels line width) served as a fixation point.

### Procedure and design

**Fig. 11:**
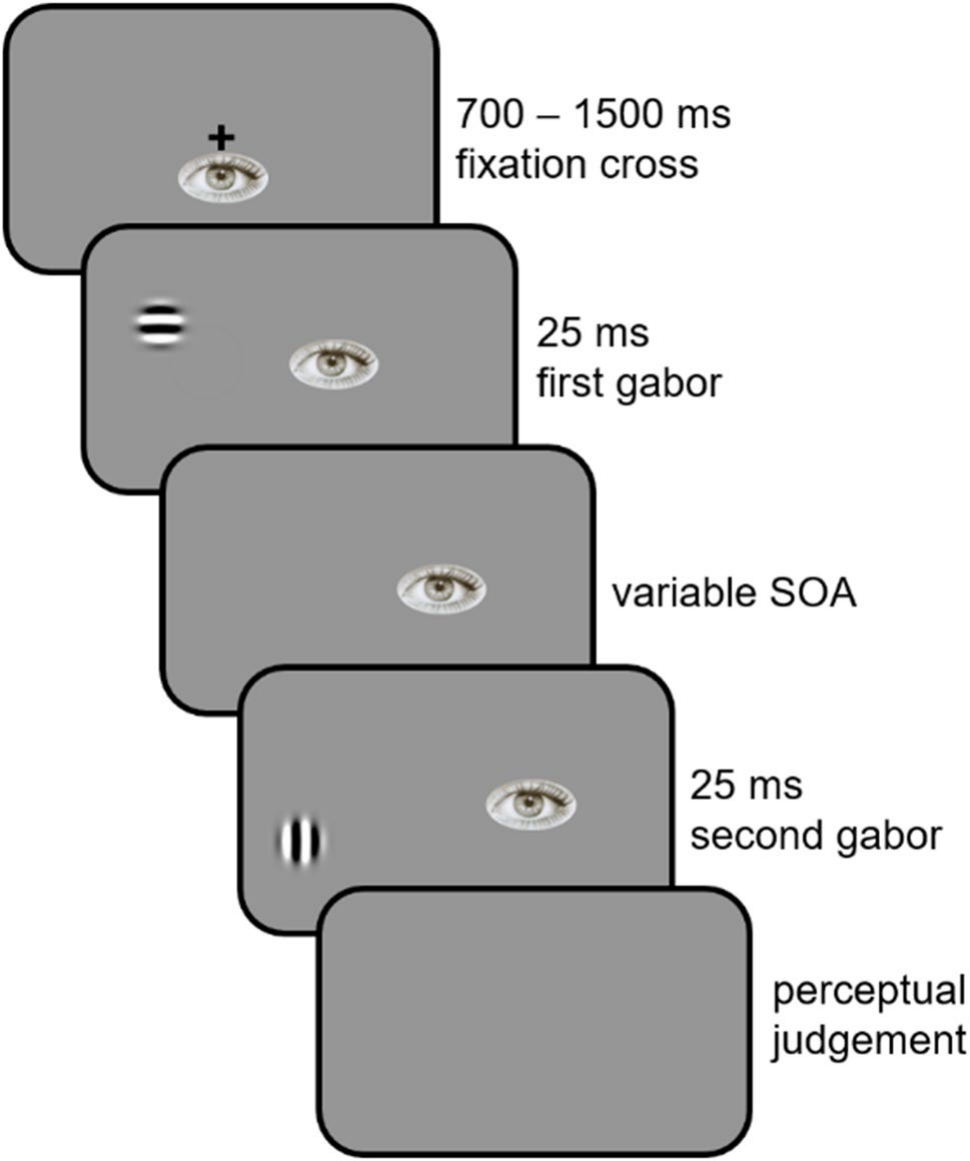
Trial structure in Experiment 3. Stimuli are not drawn to scale

A trial always started with the presentation of a fixation cross displayed at the center of the screen (Fig. 11, frame 1). After a delay of 700 to 1500 ms, the fixation cross disappeared and one of the two gabors simultaneously appeared at 14 dva eccentricity to the left, at one of two possible positions (Fig. 11, frame 2). The first position was 2 dva above the virtual horizontal line passing through the center of the screen, whereas the second was 2 dva below. After a variable stimulus onset asynchrony (SOA, Fig. 11, frame 3), the second gabor appeared at the other position (Fig. 11, frame 4). Each gabor was displayed for 25 ms. Once the second gabor had disappeared, participants had to indicate which of the vertical or horizontal gabors appeared first, by pressing the left- or right-arrow keys (Fig. 11, frame 5). The pairing between the gabor and the arrow keys was counterbalanced across participants. Participants were instructed to fixate on the fixation cross location for the entire trial and we ensured that they kept fixating on the 1.5-dva-radius area around the center of the screen. If a saccade or a drift outside this area was detected, the trial was aborted and played again later on (in the CS procedure) or immediately (in the adaptive procedures).

In the CS procedure, the set of SOAs consisted of all the possible values from 3 to 18 frames (one frame = 4.2 ms), spaced by three frames. Each SOA was randomly presented 24 times during the session. Three other SOAs of 0, 24, and 30 frames were also inserted 12 times each throughout the course of the session, for a total of 180 trials. In half of the trials, the vertical gabor was presented first, while in the other half, the horizontal gabor appeared first. In the latter case, SOAs were multiplied by −1. A null SOA corresponded to simultaneity. The positions of the gabors were counterbalanced across SOAs.

In the staircase procedure, the initial SOAs were −40, 0 and 40 frames, depending on the simple up-down track considered. In each track, after the first response reversal, the SOA changed by four frames. After the second reversal, it changed by three frames, and by two frames for the following reversals.

In the QUEST+ procedure, the set of SOAs consisted of all the possible values from −60 to 60 frames, linearly spaced by 1. The possible values for the mean were all the possible values spaced by one frame from −40 to 40 frames. Standard deviation could take values between 1 and 50 (step of 1) and lapse values between 0 and 0.08 (step of 0.01).

In the two adaptive procedures, the first gabor randomly appeared in one of the two positions.

### Results

Three outliers were removed from the analyses (based on the *Sn* method, with a criterion of 3). As in the previous experiment, an additional analysis with all participants showed that the exclusion of these outliers did not change the statistical conclusions on PSEs and JNDs, but that some correlations between estimates were artificially increased (see supplementary Tables S1 and S2).

#### Number of trials

Parameter estimations were based on 64 valid trials in the QUEST+ procedure, 180 trials in the CS procedure, and 115 trials (on average) in the staircase procedure, (range of participants’ contribution: 91−144 trials).

#### PSSs

As in the previous experiments, thresholds (PSSs here) did not vary as a function of the procedure, *F*(2,42) = 0.62, *p =* .54, and *BF*_01_ = 4.90 (see Fig. 3C). There was a significant bias when PSSs were measured with the QUEST+ (*M*_Q+_ = −1.01 frames, *SD*_Q+_ = 3.29, *range*_Q+_: −4.51 to 2.46, *BF*_01_ = 0.16 for the Bayesian one-sample *t*-test against the zero value, see also the 95% CI in Fig. 3D) and the CS procedures (*M*_CS_ = −0.96 frames, *SD*_CS_ = 2.09, *range*_CS_: −4.26 to 3.8, *BF*_01_ = 0.71), but not with the staircase one (*M*_Staircase_ = 0.41 frames, *SD*_Staircase_ = 2.38, *range*_Staircase_: −4.53 to 4.01, *BF*_01_ = 2.86).

Only the pairwise correlation between PSSs was significant for the CS and staircase procedures, *r*(19) = .46, *p* = .036. The correlation between PSSs for the two adaptive procedures and for the CS and QUEST+ procedures failed to reach significance, *r*(19) = .42, *p* = .057 and *r*(19) = .25, *p* = .27, respectively (see Table 1).

**Fig. 12:**
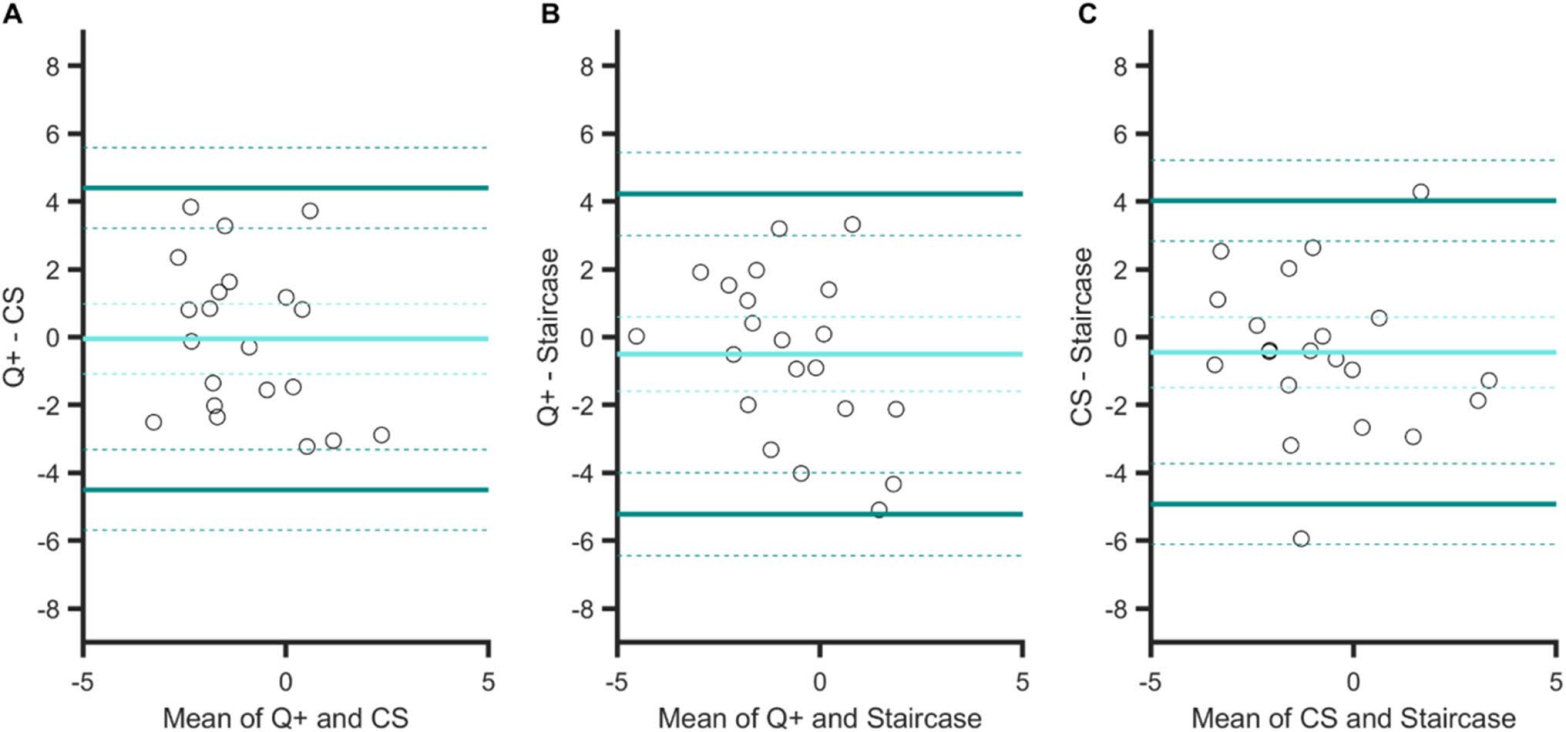
Bland–Altman plots for PSSs in Experiment 3. Agreements between: **A** the QUEST+ and CS procedures, **B** the QUEST+ and staircase procedures, and **C** the CS and staircase procedures. Light blue line: mean difference between two specific procedures (showing a possible bias for one procedure over another). Dark green lines: upper and lower limits of agreement (i.e., 1.96 × *SD* from the mean) between procedures. Dashed lines indicate 95% confidence intervals

The three Bland–Altman plots (Fig. 12) show that the degrees of agreement between procedures were similar: ranges are all about nine frames (*M* = 9.11, *SD* = 0.27). There was no bias for one procedure over another (the three 95% CIs of the mean difference contain the zero value).

#### JNDs

Unlike PSSs, sensitivity was impacted by the procedure, *F*(2,42) = 7.45, *p =* .002, marginal *R*^2^ = 0.15 (see Fig. 5C). As indicated by Tukey *HSD* post hoc analyses, measuring JNDs using the QUEST+ procedure led to smaller values (*M*_Q+_ = 4.79, *SD*_*Q+*_ = 1.73, *range*_Q+_: 1.84–8.6) in comparison with the CS procedure (*M*_*CS*_ = 7.66, *SD*_CS_ = 3.77, *range*_CS_: 2.61–14.69), *p* = .001. JNDs from the staircase procedure, however, did not differ significantly (*M*_Staircase_ = 5.88; *SD*_Staircase_ = 1.94, *range*_Staircase_: 3.31–10.55) from those measured using the QUEST+ (*p* = .12) or the CS (*p* = .20) procedures.

As for PSSs, the only significant correlation between JNDs was observed for the CS and staircase procedures, *r*(19) = .46, *p* = .035. The correlations between JNDs for the two adaptive procedures and for the CS and QUEST+ procedures did not reach significance, *r*(19) = −.14, *p* = .055 and *r*(19) = .40, *p* = .073, respectively.

**Fig. 13:**
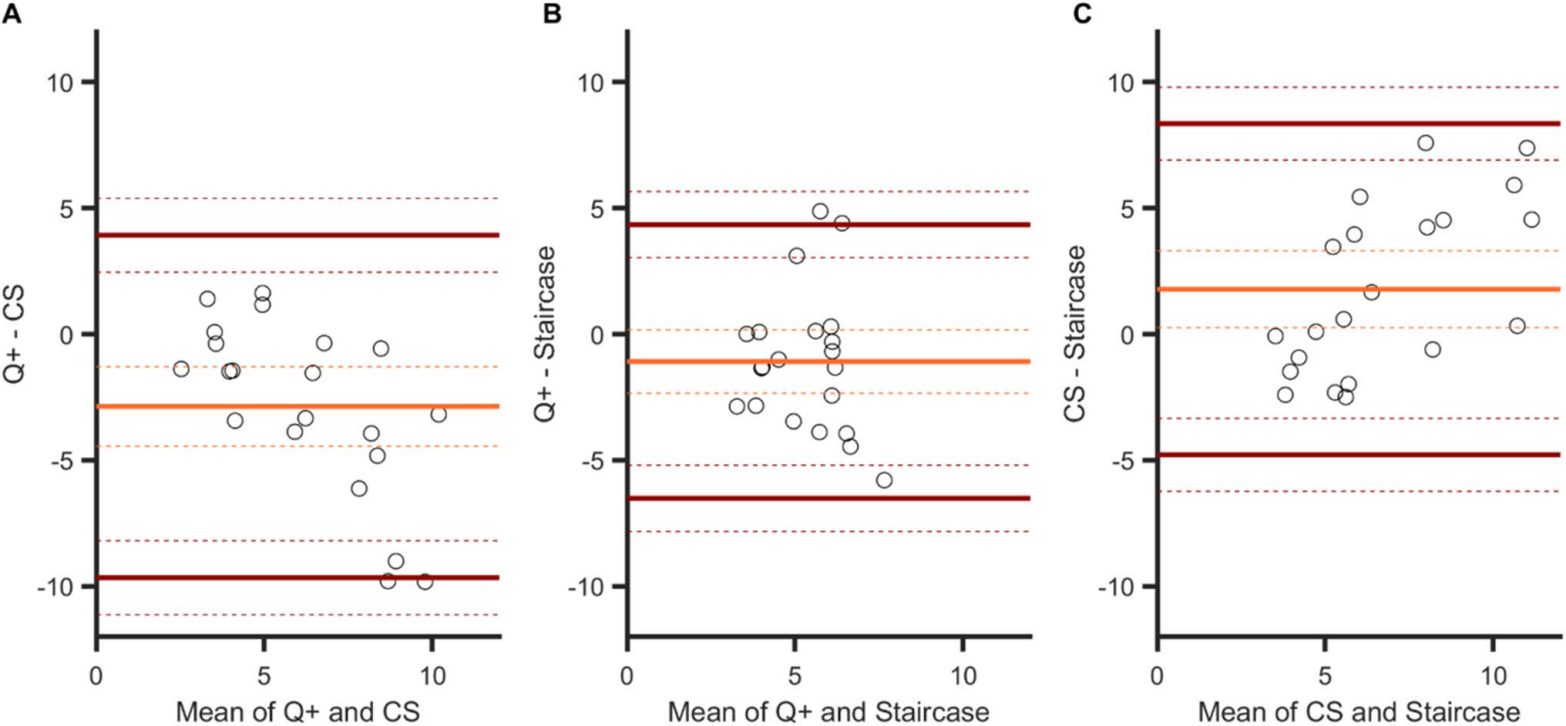
Bland–Altman plots for JNDs in Experiment 3. Agreements between: **A** the QUEST+ and CS procedures, **B** the QUEST+ and staircase procedures, and **C** the CS and staircase procedures. Light orange line: mean difference between two specific procedures (showing a possible bias for one procedure over another). Brown lines: upper and lower limits of agreement (i.e., 1.96 × *SD* from the mean) between procedures. Dashed lines indicate 95% confidence intervals

Again, the strongest degree of agreement was observed for the two adaptive procedures (agreement interval of 11 frames, [−6.5; 4.3], Fig. 13, panel B), and there was no bias between the mean differences for these two procedures. From the other two plots, we can note the presence of biases. The sensitivity to temporal order was poorer in both the QUEST+ procedure (Fig. 13, panel A) and the staircase procedure (panel C) in comparison with the CS procedure. Two linear regressions indicated that the two slopes in panels A (*a* = −1) and C (*a =* 0.85) differed significantly from zero (*p <* .001 and *p =* .002, respectively), showing that bias increased when sensitivity decreased. The two corresponding agreement intervals were comparable, 14 and 13 frames.

#### Learning effect

First, we examined whether PSSs and JNDs improved throughout the experiment, as a result of a possible learning effect (Fig. 7C). LMM confirmed that there was no effect of the block of trials on PSSs, *F*(2,42) = 1.22, *p =* .30, and *BF*_01_ = 3.2.

An improvement was however visible in terms of sensitivity: JNDs significantly decreased over the different blocks of trials, *F*(2,42) = 3.85, *p =* .029, marginal *R*^2^ = .086. Tukey *HSD* post hoc analyses revealed that there was only a trend for JNDs to be smaller in the first block of trials (*M* = 7.23, *SD =* 3.18) in comparison with both the second (*M* = 5.65, *SD =* 2.78, *p* = .067) and third blocks (*M* = 5.44, *SD =* 2.34, *p* = .054), which did not differ from each other (*p* = .10)*.*

Further analyses indicated that our participants showed a response bias; their mean PSS values differed from the zero value on the first two blocks (*BF*_01_= 0.15 and 0.69, Bayesian one-sample *t*-test). This response bias disappeared only on the last block of trials (*BF*_01_ = 3.16). Overall, mean PSS values decreased from −1.21 (*SD* = 1.86) to −0.45 (*SD* = 2.39).

## Discussion

The data from the present study obtained in 72 participants in total, tested in three different yes/no discrimination tasks that evaluated size, orientation, and temporal perception, showed that as few as 64 QUEST+ trials can provide PSE (or PSS) estimates in the same range as estimates obtained with two other psychophysical procedures commonly used in many laboratories, namely, the CS and the nonparametric staircase procedures. Overall, as expected, we observed important correlations between the PSEs of the three procedures. This study thus provides robust empirical evidence that the QUEST+ procedure, which requires a small number of trials, constitutes a reliable and efficient tool to measure perceptual thresholds and detect perceptual biases. This result is extremely important for studies interested in specific populations (e.g., in children, see Farahbakhsh et al., 2019) or for evaluating changes in perception (i.e., shifts in psychometric functions) in long experiments such as those involving pre- and post-tests.

JNDs obtained with the QUEST+ procedure were however significantly smaller than the JNDs obtained with the CS procedure in our three experiments. Despite these differences, correlations on JNDs were still high between these procedures (except for Experiment 3 on temporal perception, which will be discussed later). Since the QUEST+ procedure uses a multidimensional array of parameters to determine stimulus values to be presented, reliable slope estimation could be expected, even within 64 trials (Kaernbach, 2001; Snoeren & Puts, 1997). But before concluding that QUEST+ definitely overestimates discrimination sensitivity, it is necessary to consider that a larger number of trials could have improved the accuracy of slope estimations. This would be consistent with Kontsevich and Tyler’s (1999) work indicating that their Bayesian adaptive method, which shares many similarities with the QUEST+ procedure, needs about 300 trials to obtain a reliable slope estimation. Another way to enhance slope estimation in the QUEST+ procedure could have been to specify appropriate priors instead of using equally likely values for the psychometric function parameters. Indeed, the strength of the maximum likelihood estimation is in integrating prior information in order to constrain the research area, thereby increasing the rapidity of testing and its reliability. We decided not to use priors in our QUEST+ procedure to avoid the risk of introducing bias, even though some data by Farahbakhsh et al. (2019) suggested that QUEST+ is able to recover, at least partly, from incorrect prior information. In addition, our goal was to validate a procedure that could be easily implemented in many laboratories; determining priors requires detailed preliminary testing and statistical developments, which might limit the benefits of the QUEST+ procedure and reduce its usability.

Regarding the JNDs obtained with the staircase procedure, the other adaptive method, they did not differ from the JNDs observed in the CS procedure. By using three randomly interleaved staircase tracks, and reconstructing psychometric functions from our trial-by-trial data, we were able to obtain JND approximations comparable to those obtained with the other methods. Nevertheless, the correlations between JNDs were weaker for staircase vs. CS procedures than for QUEST+ vs. CS procedures, and were nonexistent for JNDs obtained with the two adaptive procedures. This is consistent with Alcalá-Quintana and García-Pérez (2013), who consider that any attempt to fit psychometric functions to data gathered with adaptive methods generally yields inaccurate estimates due to the lack of data, unless the procedure is explicitly designed to fulfill this goal (Kaernbach, 2001). This likely explains the stronger correlations when we considered the QUEST+ procedure. In any case, even if interleaving several staircase tracks is computationally simple, the number of trials necessary to achieve the experimental session is multiplied, which undoubtedly limits the potential benefits of the staircase procedure.

In the present study, we intended to validate the use of the QUEST+ procedure in tasks involving different perceptual dimensions. The highest correlations between PSEs were observed in Experiment 1 (size perception in fovea), with the strongest agreement between methods (see arrows in the Bland–Altman analyses in Fig. 4). In Experiment 2 (feature orientation in peripheral vision), correlations were still significant, although lower than in the preceding experiment, as was the level of agreement between procedures (CIs of the limits of agreement were clearly separated in Figs. 9 and 10). Correlations vanished in Experiment 3 (temporal perception in peripheral vision), except for CS and staircase, although these were moderate. This suggests that increasing task demands (foveal vs. peripheral stimuli discrimination and low- vs. high-level dimensions) increases the difficulty in discriminating two stimuli and adds uncertainty to the measurement of perceptual responses. Regarding JNDs, the pattern of correlations is less clear. As mentioned before, slope estimation by the staircase procedure is risky, which casts doubt on the only two significant correlations observed in Experiments 2 and 3 between CS and staircase, especially since there was no correlation in Experiment 1, where the strongest ones were expected. For the QUEST+ and CS procedures, as for PSEs, correlations between JNDs decreased between Experiments 1 and 2, to disappear in Experiment 3, as task demands increased. This is not particularly surprising, since measure uncertainty is increased in this last experiment, and it is particularly visible in the large IQR of the gold standard CS procedure (see Fig. 5C).

The increase in task difficulty also likely explains the learning effects we observed in Experiments 2 and 3, where stimuli were presented in peripheral vision. As expected from the literature (Fine & Jacobs, 2002), no rapid change in JNDs occurred in Experiment 1 on foveal perception of object size. In the other two experiments, however, performance improvement was reflected in significant decreases in JNDs that occurred over the course of the experiment. Such learning can be procedural and/or perceptual in nature, and involves both low and high-level processes (Dosher & Lu, 2017; Maniglia & Seitz, 2018). It can be extremely difficult to distinguish between these processes. Rapid improvements in perceptual performance have been reported previously (e.g., Karni & Sagi, 1993), but along with large interindividual variability, especially for stimuli presented in periphery (Beard et al., 1995; Fahle et al., 1995). In Experiment 2, the enhancement of oblique orientation discrimination is in line with previous findings (Schoups et al., 1995; Zhang et al., 2010). In particular, Zhang et al. (2010) observed improved orientation discrimination of gabors presented at 5 dva eccentricity, and learning was visible within the first 2-hour training session. In our experiments, an improvement in discrimination sensitivity was even observed over a single 1-hour session*.* In Experiment 3, performance improvement was reflected in both a decrease in JNDs and an increase in PSSs. At the beginning of the experiment, participants were more likely to respond that the vertical gabor appeared before the horizontal one (PSS < 1). Presumably, this initial bias might be due to the successive appearance of the two stimuli, one above the other, creating vertical apparent motion (which can appear for the SOAs we presented, see Wertheimer, 1912). This might have first confused participants in their responses, but at the same time, left some room for performance improvement. In our Experiment 3, contrary to a comparable study by Arstila et al. (2020), who found no learning due to a ceiling effect, we observed the disappearance of response bias across blocks of trials. The learning could be due to decreased thresholds for apparent motion. Because such improvement in apparent motion perception seems to take place only after a large number of trials (around 1000–2000 trials) in a minority of observers (Wehrhahn & Rapf, 2001), we suspect the involvement of higher-level processes in our experiment. In the end, it remains difficult to ascertain the exact nature of the learning we observed in this study, without further research (e.g., on learning specificity and durability).

As a final note, it is interesting to consider that QUEST+, like almost all adaptive procedures, targets the empirical threshold, defined as the halfway point of the psychometric function. The signal detection theory (SDT), considered as the reference frame for assessing sensitivity and bias in perceptual decision-making, provides a definition of the threshold that is independent of both the psychophysical method used and the context of the task (Green & Swets, 1966; Hautus et al., 2021; Klein, 2001). The threshold is then defined as the stimulus strength that gives a predefined level of sensitivity (e.g., *d*’= 1). Notably, some authors combined elements of SDT and Bayesian adaptive inference to determine such threshold estimates that are free of decision criterion confounds (Lesmes et al., 2015). Their methods are however restricted to detection tasks. When interested in decision criteria, it could be useful for the QUEST+ procedure to integrate this approach in any of the experimental configurations that it may allow.

## Conclusion

The present study empirically validates the QUEST+ as an efficient and quick method to determine perceptual thresholds. QUEST+ threshold estimations were as precise as the estimations obtained with the gold standard CS procedure or the widely used staircase procedure. Sensitivity estimations were however overestimated. As we suggested, this issue might be overcome by increasing the number of trials (beyond 64) and/or by using appropriate priors. QUEST+ has proved to be generalizable to different stimulus dimensions involved in perception: size, orientation, and temporality. These findings motivate us to use this Bayesian method in other psychophysical tasks and sensory modalities.

### Supplementary Information

ESM 1 (DOCX 28 kb)

## Data Availability

https://osf.io/6jkt5/?view_only=3c711fc980ee4c04a2514b41312fd2e0.
