# Supplementary material for: Empirical validation of QUEST+ in PSE and JND estimations in visual discrimination tasks
Source: Behav Res Methods. 2022 Dec 20;55(8):3984–4001. doi: 10.3758/s13428-022-02001-4 (PMC10700427; doi:10.3758/s13428-022-02001-4)
Supplement: Supplementary file 1 — (DOCX 28 kb) [file 13428_2022_2001_MOESM1_ESM.docx]

Supplementary Materials

**Table S1.** Means and Standard deviations of PSE (or PSS) and JND values obtained with the different procedures, for each experiment, when data include outliers.

|  | | Expt 1 | | |  | Expt 2 | |  | Expt 3 | |
| --- | --- | --- | --- | --- | --- | --- | --- | --- | --- | --- |
|  |  | *M* (in dva) | | *SD* |  | *M* (in °) | *SD* |  | *M* (in frames) | *SD* |
| PSE (PSS) | Quest+ | 1.57 | .07 | |  | 1.48 | 4.11 |  | -0.07 | 3.15 |
|  | Constant Stimuli | 1.56 | .07 | |  | 1.13 | 3.64 |  | -0.64 | 2.28 |
|  | staircase | 1.56 | .09 | |  | 0.22 | 3.86 |  | 0.38 | 3.94 |
| JND | Quest+ | .08 | .08 | |  | 3.02 | 1.42 |  | 5.52 | 3.13 |
|  | Constant Stimuli | .10 | .04 | |  | 4.27 | 2.45 |  | 7.75 | 3.59 |
|  | staircase | .09 | .05 | |  | 4.39 | 2.61 |  | 6.29 | 3.47 |

dva = degree of visual angle

**Table S2.** Pairwise Pearson (*r*) or Spearman (*r_s_*) correlations between PSE values and between JND values of the different procedures, for each experiment, when data include outliers. In bold, *p*-value less than .05.

|  | | Expt 1 | | |  | Expt 2 | |  | Expt 3 | |
| --- | --- | --- | --- | --- | --- | --- | --- | --- | --- | --- |
|  |  | *r* | | *p* |  | *r* | *p* |  | *r* | *p* |
| PSE (PSS) | Q+ and CS | ***r*(20) = .82** | **<.001** | |  | ***r_s_*(21) = .68** | **<.001** |  | *r_s_*(22) = .39 | .06 |
|  | Q+ and staircase | ***r*(20) = .84** | **<.001** | |  | ***r_s_*(21) = .58** | **.005** |  | ***r_s_*(22) = .56** | **.005** |
|  | CS and staircase | ***r*(20) = .80** | **<.001** | |  | ***r_s_*(21) = .58** | **.005** |  | ***r_s_*(22) = .45** | **.028** |
| JND | Q+ and CS | ***r_s_*(20) = .73** | **<.001** | |  | ***r_s_*(21) = .78** | **.005** |  | *r_s_*(22) = .38 | .07 |
|  | Q+ and staircase | *r_s_*(20) = .36 | .16 | |  | ***r_s_*(21) = .67** | **.001** |  | *r_s_*(22) = -.24 | .25 |
|  | CS and staircase | *r_s_*(20) = .37 | .41 | |  | ***r_s_*(21) = .77** | .**001** |  | ***r_s_*(22) = .46** | **.098** |
